# Metabolic syndrome and biochemical changes among non-alcoholic fatty liver disease patients attending a tertiary care hospital of Nepal

**DOI:** 10.1186/s12876-018-0843-6

**Published:** 2018-07-06

**Authors:** Bashu Dev Pardhe, Shreena Shakya, Anjeela Bhetwal, Jennifer Mathias, Puspa Raj Khanal, Roshan Pandit, Jyotsna Shakya, Hari Om Joshi, Sujan Babu Marahatta

**Affiliations:** 1 Department of Laboratory Medicine, Manmohan Memorial Institute of Health Sciences, Kathmandu, Nepal; 2 Department of Health Science, National Open College, Sanepa, Lalitpur, Nepal; 3 Department of Radiology, Bhaktapur District Hospital, Bhaktapur, Nepal; 4 Department of Public Health, Manmohan Memorial Institute of Health Sciences, Kathmandu, Nepal

**Keywords:** Non-alcoholic fatty liver disease, Transaminases, Metabolic syndrome, Biochemical markers

## Abstract

**Background:**

Non-alcoholic fatty liver disease (NAFLD) is mutually and bidirectionally linked with metabolic syndrome (MetS) of which it is both the cause and the consequences. Worldwide, 6.3 to 33% of the general populations are estimated to suffer from the disease with even higher prevalence in the group sharing metabolic co-morbidities. Hence, this study aims to recognize various risk factors including metabolic components and blood parameters to predict the possible incidence of the disease.

**Methods:**

Total of 429 (219 NAFLD and 210 control) subjects were conveniently selected for study during the period of 9 months. Diagnosis of non-alcoholic fatty liver disease was done by liver imaging and based on liver enzymes. Assessment of metabolic syndrome was done by International Diabetic Federation (IDF) and National Cholesterol Education Program Adult Treatment Panel III (NCEP ATP III) criteria. All biochemical and hematological parameters and liver enzymes were estimated by using standard guideline. Mean comparison of quantitative data in different groups were performed using analysis of variance (one-way ANOVA). Risk estimation of NAFLD associated with each character was verified by Chi-square test.

**Results:**

There was significant high levels of body mass index (BMI), waist circumference (WC) and lipid profiles in NAFLD patients in comparison to control population (*p* < 0.001). Further, according to the NCEP ATP III criteria, 13.6% of NAFLD were present with MetS where risk estimate was significant (OR = 2.15). Whereas, other criteria (IDF) for MetS showed higher frequency (30.1%) with higher risk (OR = 29.75) for the presence of MetS in NAFLD patients. The change in triglycerides (TG) and HDL-C (high density lipoprotein cholesterol) was also statistically significant in different grades of NAFLD. High risk for NAFLD was associated with existing co-morbid conditions like cardiovascular risk patients (3.18 times) followed by obese patients (1.72 times) and Diabetes Mellitus patients (1.68 times) at a significant level.

**Conclusion:**

The result of this study suggests that there is an increased prevalence of all the components of MetS and significant changes in biochemical markers in cases of NAFLD. Timely diagnosis would help in delaying its complications and co-morbidities.

## Background

Nonalcoholic fatty liver disease (NAFLD) was described almost 6 decades ago and has emerged as the most common form of the chronic liver disease and its prevalence is likely to continue rising [1, 2]. Worldwide, 6.3 to 33% of the general population are estimated to suffer from the disease with even higher prevalence in the group sharing metabolic co-morbidities [3].

Hepatic steatosis based on either imaging studies or liver biopsy confirms NAFLD in patients with clinical signs and symptoms without the considerable abuse of alcohol (< 20 g ethanol/ day) [4]. The pathological spectrum of this disease ranges from a simple steatosis to steatohepatitis, fibrosis or cirrhosis of the liver [5]. Underlying metabolic risk factors for the disease progression include old age (> 50 years), sex (male> female), central obesity, insulin resistance (IR), Type 2 diabetes mellitus (T2DM), increased ferritin levels and genetic polymorphisms (patatin-like phospholipase domain-containing 3 (PNPLA3) I148M polymorphism) [6].

Previously, NAFLD has been considered as a hepatic component of metabolic syndrome (MetS), [7] but recently an association between NAFLD and MetS in type 2 diabetes mellitus has been described but the phenomenon is very complex. Indeed NAFLD is mutually and bidirectionally linked with MetS of which it is both the cause and the consequences. [2] Tan et al*.* [8] suggested International Diabetic Federation (IDF) criteria is applicable in an Asian population for risk assessment of MetS while Pokharel et al [9] suggests National Cholesterol Education Program Adult Treatment Panel III (NCEP ATP III) to be specific for MetS in our population but not applicable.

NAFLD progresses as a silent disease which is disclosed only after a routine health check-up following elevated transaminases levels with no any other recognized causes of fatty liver like alcohol, virus, drugs, autoimmunity [10, 11]. Liver biopsy remains the gold standard method for the diagnosis of hepatic steatosis and helps in exclusion from secondary etiology of liver injuries like drug-induced hepatotoxicity, Wilson disease and autoimmune hepatitis [4, 12]. Histological examination also differentiates NAFLD into its sub-stages; non-alcoholic fatty liver (NAFL) and non-alcoholic steatohepatitis [3, 13, 14]. The development of new technology has given the alternatives to screen the patients without much of inconvenience like ultrasonography, computed tomography, magnetic resonance imaging, transient elastography [15]. Furthermore, semiquantitative ultrasound score i.e. ultrasonographic fatty live indicator (US-FLI) is more specific for metabolic/histological variables in NAFLD [16]. Due to cost-effectiveness and availability of ultrasonography, it is widely used to detect and grade NAFLD in our country.

Various changes in the biochemical profile can be observed in the patients with the disease. Elevated serum transaminases level remains the most common or sometimes the only abnormal laboratory finding. Although the prime abnormality, liver enzymes may be normal in greater than 70% of the patients with NAFLD [4]. Serum level of alkaline phosphatase (ALP), γ-glutamyl transferase (GGT) or both is frequently elevated although the level is lower than in alcoholic hepatitis. There is also an increase in the triglyceride (TG) and low-density lipoprotein cholesterol (LDL-C) level posing a cardiovascular risk [17].The mechanisms underlying excess cardiovascular risk in NAFLD patients is much more complex and involves both generic mechanisms associated with the MetS and other specifically associated with NAFLD [2, 18]. The studies from the past decade have projected the increasing morbidity and mortality of the patients with NAFLD, not due to the liver-related complications but primarily because of cardiovascular disease [19].

Early diagnosis is very important in the timely management of the NAFLD. But there is not a single biochemical marker for confirmation of NAFLD. Hence, this study aims to recognize various risk factors including metabolic components and blood parameters to predict the possible incidence of the disease.

## Methods

A hospital-based cross-sectional study was conducted in a tertiary care teaching hospital in Nepal during the period of 9 months. A total number of 429 (219 NAFLD and 210 control) subjects were selected conveniently for the study from the patients visiting for their regular medical checkup. Diagnosis of NAFLD was based on imaging (fatty liver) in the absence of competing for the cause of steatosis [12, 20]. Fatty live imaging by ultrasonography was performed by experienced Radiologist by Medison SonoAce R7 ultrasound machine and patient with visual diffused hepatic steatosis were further graded to describe the extent of fatty change in the liver. Grading was done based on the standard criteria accepted by American, Gastroenterological Association [21].

Grade I: Increased hepatic echogenicity with visible periportal and diaphragmatic echogenicity.

Grade II: Increased hepatic echogenicity with imperceptible periportal echogenicity, without obscuration of the diaphragm.

Grade III: Increased hepatic echogenicity with imperceptible periportal echogenicity and obscuration of the diaphragm.

Patients with normal hepatic ultrasonography were categorized as a control. Information regarding the patient demography (age, sex), height, weight, blood pressure, and related drug therapy were collected, measured by standard protocol and recorded in a clinical profile form. About 5 ml of fasting blood specimen was collected from every individual, processed and then analyzed for blood chemistry parameters (lipid profile, liver profile and renal profile) by standard methods as per the guideline provided by the reagent manufacturer (Human Gm Bh, Germany). Fasting blood glucose was estimated to diagnose Diabetes Mellitus. For categorization of DM from the total population, the T2DM diagnostic criteria provided by the International Diabetes Federation (IDF) was used [22]. Further, the study population was categorized as with and without metabolic syndrome based on the cut-offs value provided by National Cholesterol Education Adult Treatment Panel III (NCEP/ATP III) criteria 2001 and International Diabetic Federation (IDF) criteria 2005 [23]. Total cholesterol (TC), TG, and high density lipoprotein cholesterol (HDL-C) were estimated and LDL-C was calculated by using Friedwald equation. Aspartate aminotransferase (AST), alanine aminotransferase (ALT), ALP, total protein, and albumin were estimated in the total population to assess liver function by using standard methods. The normal range for both ALT and AST was considered up to 42 U/L in male and up to 32 U/L in female as provided by reagent manufacturer guideline [24]. All biochemical parameters were analyzed by HumaStar 300 fully automated analyzer following manufacturers’ instructions. Hematological parameters were analyzed by automated cell counter (Huma Count 30^TS^ - HUMAN DIAGNOSTICS).

### Inclusion and exclusion criteria

Patients above the age of 30 years and below 60 years attending Manmohan Memorial Teaching Hospital (MMTH) during the period of 9 months were included in the study after taking their informed and written consent.

Control- Apparently healthy individuals visiting for their regular health checkup and without any history of elevated liver enzymes during past 6 months and without fatty liver (imaging) on diagnosis were included.

NAFLD- Patients with a history of elevated liver enzymes at least once during past 6 months of their hospital visit were included and evaluated by imaging (fatty liver) for the confirmation of NAFLD. The population with a history of regular alcohol intake in past 6 months, history of steroid intake for > 2 weeks in past 6 months and any evidence of prescribed hepatotoxic drugs (methotrexate) were excluded from the study. In addition Patients with liver cirrhosis, kidney disease, evidence of bone diseases were also excluded from the study. Further, patients with positive Hepatitis B surface antigen and positive hepatitis C antibody on blood test were also barred.

Ethical approval was taken from the Institutional Review Committee (IRC) of Manmohan Memorial Institute of Health Sciences (MMIHS).Written consent was taken from each individual before their participation in the study. Data regarding personal information were coded and kept confidential.

### Data analysis

Data were analyzed using SPSS version 20.0 (IBM Corp., Armonk, NY, USA) and Microsoft Excel 2013. Quantitative data recorded with normal distribution were expressed in mean ± SD and were analyzed by Student’s t-test. Mean comparison of quantitative data in different groups were performed using analysis of variance (one-way ANOVA). Qualitative data were analyzed by Chi-square test. Risk estimation of NAFLD associated with each character was also verified by Chi-square test. *P* < 0.05 was considered statistically significant.

## Results

The study was carried out in Manmohan Memorial Teaching Hospital (MMTH) among 429 study population of which 225 were male and 204 were female. The mean age of study population was 56 ± 10 year. On diagnosis by ultrasonography, 54% were present in grade I, 39% with grade II and 7% were with grade III NAFLD. On average, AST exceeded the upper normal limit in 46% of cases, ALT in 54%, ALP in 9% and GGT in 23%. There were significant high levels of body mass index (BMI), waist circumference (WC), systolic blood pressure (SBP), and diastolic blood pressure (DBP) in NAFLD patients as compared to control population (*p* < 0.001). In addition, lipid profile parameters showed significant statistical elevation among NAFLD patients. (Table 1).

**Table 1:** Comparisons of biochemical parameters between control and NAFLD populations

	NAFLD	Control	*p*
Age (years)	44.24 ± 13.45	45.38 ± 14.67	0.985
BMI (kg/m^2^)	26.41 ± 4.74	23.61 ± 3.51	**< 0.001**
WC (cm)	89.55 ± 9.73	76.34 ± 6.12	**< 0.001**
SBP (mmHg)	133.79 ± 12.72	126.64 ± 10.13	**< 0.001**
DBP (mmHg)	85.82 ± 7.91	80.85 ± 7.01	**< 0.001**
Total Bilirubin (mg/dl)	0.85 ± 0.162	0.85 ± 0.097	0.776
Direct Dilirubin (mg/dl)	0.24 ± 0.081	0.23 ± 0.047	0.619
ALT (U/L)	41.93 ± 23.98	22.86 ± 6.99	**< 0.001**
AST (U/L)	38.66 ± 20.20	25.64 ± 7.27	**< 0.001**
ALP (U/L)	170.74 ± 51.39	143.62 ± 40.79	**< 0.001**
Uric Acid (mg/dl)	5.34 ± 1.18	4.64 ± 1.08	**< 0.001**
FBS (mg/dl)	106.52 ± 37.82	89.23 ± 16.43	**< 0.001**
Total cholesterol (mg/dl)	191.62 ± 40.0	154.51 ± 18.9	**< 0.001**
Triglyceride (mg/dl)	191.30 ± 94.29	113.83 ± 21.56	**< 0.001**
HDL-C (mg/dl)	42.51 ± 3.50	46.18 ± 3.82	**< 0.001**
LDL-C (mg/dl)	110.82 ± 35.79	85.57 ± 18.76	**< 0.001**
Creatinine (mg/dl)	0.93 ± 0.16	0.92 ± 0.16	0.561
Urea (mg/dl)	25.41 ± 5.96	27.014 ± 6.63	0.13
Hemoglobin (g/dl)	14.47 ± 1.83	17.46 ± 2.21	0.25
TLC (cells/μl)	8895.89 ± 1226.22	5931.43 ± 1003.12	0.43
RBC (millions/ μl)	4.98 ± 0.69	4.83 ± 0.52	0.145
Platelet (cells/ μl)	282,000.01 ± 72,872.61	297,442.86 ± 69,208.074	0.196
Total protein (g/dl)	6.49 ± 0.65	7.38 ± 0.65	**< 0.001**
Albumin (g/dl)	3.42 ± 0.41	4.41 ± 0.53	**< 0.001**

Bold represents statistically significant values

*BMI* body mass index, *WC* waist circumference, *SBP* systolic blood pressure, *DBP* diastolic blood pressure, *ALT* alanine aminotransferase, *AST* aspartate aminotransferase, *ALP* alkaline phosphatase, *FBS* fasting blood sugar, *HDL-C* high density lipoprotein cholesterol, *LDL-C* low density lipoprotein cholesterol, *TLC* total leucocyte count, *RBC* red blood cell count

Overall, distributions of metabolic components in NAFLD and control groups are illustrated in Table 2. The varied frequency distribution of metabolic components was reported as, low HDL-C with the highest frequency (69.8%), and followed by high TG (60.27%), overweight (57.5%) and hypertension (56.1%). Further, according to the NCEP ATP III criteria, 13.6% of NAFLD were present with MetS where risk estimate was significant (OR = 2.15). In addition, other criteria (IDF) for MetS showed higher frequency (30.1%) with higher risk (OR = 29.75) for the presence of MetS in NAFLD patients.

**Table 2:** Distribution of metabolic components in study population

Metabolic components	Control	NAFLD	X^2^	OR (95%CI)
Age: > 50 years	10%	16.4%	1.28	0.56(0.21–1.53)
BMI: ≥25 kg/m^2^	30%	57.5%	10.99	3.16(1.58–6.31)^a^
WC: > 94 cm (M),> 80 cm (F)	11.4%	56.1%	31.75	0.10(0.04–0.24)^a^
WC: > 102 cm (M),88 cm (F)	1.4%	30.1%	21.821	29.76(3.88–228.03)^a^
Blood Pressure: ≥130/85 mmHg	35.7%	56.1%	6.01	2.30(1.17–4.50)
TG: ≥150 mg/dl	2.9%	60.27%	53.98	51.58(11.71–227.12)^a^
Low HDL: < 40 mg/dl(M),< 50 mg/dl (F)	47.1%	69.8%	7.61	2.59(1.30–5.16)^b^
MetS + (NCEP ATPIII-criteria)	1.4%	13.6%	10.31	2.15(1.70–2.52)^b^
MetS + (IDF-criteria)	1.4%	30.1%	21.82	29.75(3.888–228.03)^a^

^a^-*p* < 0.001, ^b^-*p* < 0.005

*F* female, *M* male, *MetS + (NCEPATPIII)* metabolic syndrome present by National Cholesterol Education Program Adult Treatment Panel III, *MetS + (IDF)* metabolic syndrome present by International Diabetic Federation

When variations in liver enzymes were compared in different grades of NAFLD, it was observed that with an increase in hepatic steatosis, ALP level also increased significantly. Also, changes in ALT were significant between the grades of NAFLD while no significant change in AST was observed. The change in TG and HDL-C was also statistically significant in different grades of NAFLD where TG was found to increase while HDL-C decreased with steatosis. There was no significant change in TC and LDL-C between different grades of NAFLD-(Table 3).

**Table 3:** Comparison of liver markers and lipid profile between different grades of NAFLD

Liver markers	Grade I- NAFLD (*n* = 120)	Grade II- NAFLD (*n* = 87)	Grade III- NAFLD (*n* = 12)	p
ALT (U/L)	33.65 ± 16.51	53.52 ± 29.20	40.75 ± 9.42	**0.002**
AST (U/L)	34.87 ± 20.63	43.86 ± 19.37	38.75 ± 17.53	0.191
ALP (U/L)	160.80 ± 51.28	177.48 ± 44.57	221.25 ± 74.10	**0.049**
TP (g/dl)	6.70 ± 0.57	6.26 ± 0.70	6.20 ± 0.47	**0.014**
Albumin (g/dl)	3.53 ± 0.42	3.31 ± 0.38	3.07 ± 0.13	**0.021**
TB (mg/dl	0.85 ± 0.16	0.82 ± 0.09	1.07 ± 0.36	**0.011**
DB (mg/dl)	0.23 ± 0.06	0.24 ± 0.10	0.27 ± 0.15	0.593
Lipid profile				
TC	184.21 ± 36.67	197.71 ± 41.05	221.51 ± 53.35	0.117
TG	156.97 ± 62.95	227.86 ± 112.32	269.57 ± 85.23	**0.001**
HDL-C	43.62 ± 2.87	41.34 ± 3.99	40.00 ± 2.16	**0.009**
LDL-C	109.18 ± 32.48	110.71 ± 38.44	127.51 ± 53.25	0.627

Bold represents statistically significant values (*p* < 0.05). n- the number of patients

The relative risk of co-morbidity along with NAFLD is demonstrated in Table 4. High risk for NAFLD was associated with existing co-morbid conditions like cardiovascular risk patients (3.18 times) followed by obese patients (1.72 times) and Diabetes Mellitus patients (1.68 times) at a significant level. (Table 4).

**Table 4:** Risk estimation for NAFLD in association with pre-existing co-morbid conditions

	NAFLD	Control	X^2^	RR	*p*
Diabetes (FBS ≥ 126 mg/dl)	(n)	(n)			
Yes	36	9	5.621	1.68	0.016
No	183	201			
Cardiovascular Risk (TC/HDL-C > 5)					
Yes	135	9	52.721	3.18	**< 0.001**
No	84	201			
Hyperurecemia (UA > 7.5 mg/dl)					
Yes	33	15	2.259	1.41	0.107
No	186	195			
Central Obesity (WC > 94 cm(M),> 80 cm(F))					
Yes	123	24	34.909	2.50	**< 0.001**
No	96	186			

Bold represents statistically significant values (*p* < 0.05). RR- relative risk, n- the number of population

## Discussion

In our study, the overall incidence of NAFLD was about 51%. The overall prevalence of NAFLD was reported in Western countries, ranging from 15 to 51%. However, Asian countries have reported lowest prevalence of NAFLD, for instance, Japan’s prevalence rate ranged from 9 to 14% [25]. Elevated liver enzymes (ALT, GGT, AST) are the sign of liver injury and may be the potent surrogate markers of NAFLD [26]. In the present study, there was a significant rise in the liver enzymes and lipid profiles except for HDL-C, which was significantly less as compared to the controls. Likewise, a study carried out by Agarwal et al. and Uttareshvar et al also reported an increase in TG, TC, VLDL-C, LDL-C and decrease HDL-C levels, indicating possible atherogenic dyslipidaemia [27, 28] . Thus, most of the earlier studies are in concordance with our observation. The influx of high fatty acids in the liver causes liver toxicity and additionally, inflammatory cytokines, TNF-6 also plays a major role in the development of hepatocellular injury causes NAFLD and fatty liver with mild to moderate increase of liver enzymes [29].

Noteworthy, important and well-established clinical association of NAFLD with dyslipidaemia, hypertension, and obesity has been documented in several studies resulting in increased mortality rates in many countries. In our study, low HDL-C and hypertriglyceridaemia are present in 69.8 and 60.27% of NAFLD patients respectively which was in accordance with the study done by Santhoshakumari et al*.* [30]. Additionally, the prevalence of low HDL-C was about 71.7% and hypertriglyceridaemia was about 42.4% in a study by Rafique et al [31]. The probable reason for the high incidence of dyslipidaemia might be due to unhealthy diet and lack of exercise. Similarly, the present study showed that over-weight was present in 57.5% and hypertension in 56.1% of NAFLD patients which was higher compared to the study of Santhoshakumari et al and Shen et al [30, 32].

The overall prevalence of metabolic syndrome in NAFLD patients varied based on the diagnostic criteria used (IDF, NCEP ATP III). In the present study, the prevalence of MetS was highest (30.1%) with the IDF criteria, showing higher risk (OR = 29.75, 95%CI: 3.88–228.03). In the study of Chen et al*.*, the prevalence of MetS was 11.11, 8.48 and 5.30% on the basis of diagnostic criteria IDF, NCEP ATP III and Chinese Diabetes Society (CDS) respectively [33]. This difference might be due to the population sample studied and the diagnostic criteria used. The lower prevalence (13.6%) of NCEP ATP III criteria is due to its relatively higher cut off values for waist circumference and this can underestimate the prevalence of MetS and risk of CVD in our population [34].

In this study, the majority of patients (54.8%) had grade I NAFLD. When liver enzymes were compared in different grades of NAFLD, ALP level increased significantly with increase in hepatic steatosis. Further, changes in ALT were also significant between the grades of NAFLD while no significant change in AST was observed. To the flip side, Cordeiro et al reported no significant changes in ALT, AST, and ALP levels among individuals with hepatic steatosis [35]. In the current study, the change in TG and HDL-C was also statistically significant in different grades of NAFLD reflecting increased TG but decreased HDL-C levels with steatosis. No any significant changes in TC and LDL-C were noted between different grades of NAFLD. In contrast to our study, the differences in serum TG and TC were not statistically significant with the increasing grades of NAFLD but statistically significant lower serum HDL-C was observed in the study of Kirovski et al. [36]. Furthermore, in the study of Mahaling et al., the change in TC, HDL-C, LDL-C, and VLDL-C showed statistical significance with increasing grades of NAFLD (*P* < 0.05), yet TG showed no significance change [27].

We also observed the higher risk of developing NAFLD with existing co-morbid conditions like cardiovascular risk, obesity and Diabetes Mellitus. In our study, cardiovascular risk patients had 3.18 times the risk of having NAFLD as compared to the control group. Incongruous, a meta-analysis study conducted in 2011 reported that the patients with NAFLD had a twofold higher risk of CVD than the control population [37]. Likewise, the obese group showed 1.72 times the risk in our setting, whereas, from a study by Khadka B et al., overweight and obese groups had 4.2 and 5.1 times the risk of having fatty liver, respectively, as compared to their normal counterparts [38].

Moreover, insulin resistance in addition to chronic dyslipidaemia appears to be a crucial mechanism of NAFLD. There is evidence that NAFLD is highly prevalent in patients with diabetes mellitus and increasing evidence suggests that diabetic patients are at high risk for developing NAFLD [10]. In addition, T2DM increases the risk of developing liver-related death by up to 22-fold as well as overall death by 2.6–3.3-fold in patients with NAFLD [4].

This study provides insight on the relationship between NAFLD and MetS and risk of co-morbidity in NAFLD patients in a specific geographical area, which could be wothful in monitoring and management of NAFLD. This was time framed cross-sectional study in a small setting with relatively lower sample size. Further, well-designed follow-up studies are needed to elucidate the causative relationship between NAFLD and MetS. The diagnosis of NAFLD was only based on imaging of hepatic steatosis and further fibrosis and cirrhosis were not confirmed by liver biopsy. Diagnosis of MetS was based on broad clinical criteria’s proposed by NCEP ATP III and IDF with fulfilling three minimum components, but there may be multiple clinical and biochemical presentation on the different clustering of risk factors. Hence, the optimal defining criteria need to be followed in future studies.

## Conclusion

The result of this study suggests that there is an increased prevalence of all the components of MetS and significant changes in biochemical markers in cases of NAFLD. Therefore, whenever metabolic components are encountered in the clinical setting, patients must be evaluated for the diagnosis of NAFLD by imaging (fatty liver). Furthermore, incessant endeavors are essential to study the prevalence of NAFLD within the population to monitor the epidemiology of this disease. Timely diagnosis would help in delaying its complications and also play a major role in preventing cardiac diseases as its association with metabolic syndrome is frequent.

## Abbreviations

ALP: Alkaline Phosphatase
ALT: alanine aminotransferase
AST: aspartate aminotransferase
BMI: body mass index
CDS: Chinese Diabetes Society
CVD: cardiovascular disease
FBS: fasting blood sugar
HDL-C: high density lipoprotein cholesterol
IDF: International Diabetic Federation
IR: insulin resistance
LDL-C: low density lipoprotein cholesterol
MetS: metabolic syndrome
NAFLD: non-alcoholic fatty liver disease
NCEP/ATP III: National Cholesterol Education Program Adult Treatment Panel III
PNPLA: patatin-like phospholipase domain-containing 3
T2DM: type 2 Diabetes Mellitus
TC: total cholesterol
TG: triglyceride
TNF: tumor necrosis factor
WC: waist circumference
